# MaxEnt Modeling for Predicting the Potential Geographical Distribution of *Hydrocera triflora* since the Last Interglacial and under Future Climate Scenarios

**DOI:** 10.3390/biology13090745

**Published:** 2024-09-22

**Authors:** Qitao Su, Zhixuan Du, Yi Luo, Bing Zhou, Yi’an Xiao, Zhengrong Zou

**Affiliations:** 1 College of Life Sciences, Jiangxi Normal University, Nanchang 330022, China; suqitao@jgsu.edu.cn; 2 School of Life Sciences, Jinggangshan University, Ji’an 330049, China

**Keywords:** species distribution model, climate change, potentially suitable areas, distribution centroid, China

## Abstract

**Simple Summary:**

*Hydrocera triflora* is primarily distributed across southeastern and southern Asia and is classified as endangered within China. The impact of climate change on the spatial distribution of this species remains uncertain. In this study, we employed the MaxEnt model to examine the migration patterns of *H. triflora* under the influence of climate change. Our findings indicate that the current potential distribution area is more extensive than during the three historical periods analyzed. Furthermore, projections suggest that the potential habitable area for *H. triflora* will continue to expand to higher altitudes by the year 2100. It is advised that protected areas be established within the current core distribution regions. Concurrently, public awareness and educational initiatives should be enhanced to mitigate the impact of human activities on wild populations. Strengthening the conservation of wild resources is imperative.

**Abstract:**

*Hydrocera triflora* is a perennial herb found in southeastern and southern Asia. In China, it is only found in Hainan Province. With global climate change, studying the impact of climate change on the distribution of *H. triflora* can provide a theoretical basis for the scientific protection of this species. In this study, the MaxEnt model was used to predict the potential distribution area of *H. triflora* in China under historical, current, and future periods based on 66 distribution points and 12 environmental variables. The results were as follows: (i) The main environmental variables affecting the distribution of *H. triflora* were precipitation in the coldest month and in the wettest quarter, with elevation also being a significant factor. (ii) Over the past three periods, the last interglacial, last glacial maximum, and mid-Holocene, the suitable area for *H. triflora* initially decreased and then increased. The suitable area reached the lowest value in the last glacial maximum period, at only 27.03 × 10^4^ km^2^. (iii) The current potential distribution area is 67.81 × 10^4^ km^2^, and the optimal area is mainly distributed in the Guangxi, Guangdong, and Hainan provinces. (iv) Under future climate scenarios, the potential distribution area of *H. triflora* is projected to increase by 11.27~90.83 × 10^4^ km^2^. It is expected to reach a maximum value (158.64 × 10^4^ km^2^) in 2081~2100 under the SSP-585 climate scenario, with the distribution centroid shifting to higher latitudes. The newly gained optimal habitats will provide potential areas for introduction and ex situ conservation of this species.

## 1. Introduction

Climate change is becoming an increasingly important issue that deserves our attention [1,2]. According to a relevant report released by the United Nations Intergovernmental Panel on Climate Change (IPCC), temperatures are expected to keep rising in the future due to global warming [3]. Long-term field monitoring data indicate that global warming may significantly influence the geographic distribution patterns of species, with effects differing among species [4,5]. For example, climate warming has led to an upward shift in the elevation range of European beech trees (*Fagus sylvatica*) by 70 m in the Montreal Mountains of Spain over the past 55 years, resulting in their replacement by lower-elevation species in their original habitats [6]. Similarly, in the Qinling Mountains of China, the upper limit of the distribution elevation of *Larix chinensis* has increased by 24.7 m, while the distribution elevation of *Abies fargesii* and *Betula albosinensis* has exhibited only minor changes [7].

In addition to long-term field records, species distribution models (e.g., MaxEnt [8,9], GARP [10,11], GAM [12], GLM [13]) can be employed to assess the impact of climate change on species distribution patterns [4,14,15]. The MaxEnt model predicts the distribution of a specific species within a given area by establishing a relationship between environmental variables and species distribution [16]. Compared to other species distribution models, the MaxEnt model is currently considered an ideal predictive tool. Due to its high precision, it can generate more accurate results even with limited species distribution data [9,17].

*Hydrocera triflora* is an aquatic herb belonging to the genus *Hydrocera*, which is a monotypic of the family Balsaminaceae. Although it is not considered endangered according to the IUCN categories, it is endangered in China. It was listed by the Ministry of Ecology and Environment of the People’s Republic of China as regionally extinct (RE) in 2013 [18], but was reclassified as endangered (EN) in 2023 [19]. There is an urgent need for targeted conservation efforts. Additionally, *H. triflora* exhibits primitive characteristics, such as free petals and a fleshy, indehiscent pseudoberry fruit, and is adapted to aquatic environments [20]. These traits are of significant importance for studying the origin, floral evolution, and adaptive differentiation within the Balsaminaceae family. Currently, research on *H. triflora* has predominantly concentrated on genomics [21], morphological structure [22], and chemical composition [23], among other aspects. However, investigations into the conditions of its wild populations remain limited. Over time, the habitat of *H. triflora* has diminished due to escalating land development demands, human activities, and various economic development initiatives. Studying the distribution patterns of *H. triflora* is essential for enhancing the exploration and conservation of its wild populations.

In this study, the MaxEnt model was employed to analyze the potentially suitable habitats for *H. trifloral* in China during the last interglacial (LIG), last glacial maximum (LGM), and mid-Holocene (MH) periods, as well as under three future climate scenarios, namely, the shared socioeconomic pathways (SSP-126, SSP-245, SSP-585), across four time periods (2021–2040, 2041–2060, 2061–2080, and 2081–2100). The research objectives were as follows: (i) to identify the potential distribution area of *H. triflora* in the current period and determine the environmental factors influencing its distribution; (ii) to analyze the potential distribution area for *H. triflora* under historical and future climate change scenarios; and (iii) to assess the changes in potential habitat distribution across historical, current, and future periods. It is crucial to utilize scientific research on the potential habitats of this plant for the successful reintroduction and restoration of the species.

## 2. Materials and Methods

### 2.1. Distribution Data Acquisition and Processing

**Figure 1:**
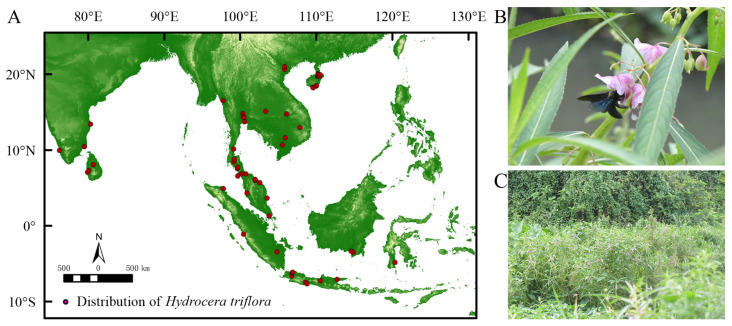
Current spatial distribution and habitat of *H. triflora.* ((**A**): Current spatial distribution of *H. triflora*; (**B**): *Xylocopa* sp. Major pollinator of *H. triflora*; (**C**): The habitat of *H. trifloral* in Hainan Province).

The global distribution data of *H. triflora* were obtained from GBIF (https://www.gbif.org/, accessed on 3 March 2023), NSII (http://nsii.org.cn/, accessed on 3 March 2023), PPBC (http://ppbc.iplant.cn/, accessed on 3 March 2023), and other websites and from academic papers from CNKI [24], as well as field data. A total of 145 records were collected, with 64 recorded in GBIF. The NSII contributed 12 records, while the PPBC provided 54 records. Additionally, 13 records were sourced from CNKI and 2 records were obtained through on-site investigations. From the PPBC distribution records, the Baidu Map coordinate pick-up system (https://api.map.baidu.com/lbsapi/getpoint/index.html, accessed on 4 March 2023) was used to upload the location to obtain the distribution of the species in terms of longitude and latitude. For the distributed data obtained from the GBIF, NSII, and academic papers of the CNKI, invalid information such as errors, noncoordinate points, and duplicate points was removed. To avoid data overfitting, ENM Tools was used to filter all distribution data and divide the map into 1 × 1 m grids, and only one distribution record was reserved in each grid. The distribution data for *H. triflora* were acquired through multiple methodologies, resulting in the identification of 17 distribution records within China. To improve the accuracy of the predictive models, global distribution data for *H. triflora* were also incorporated. A total of 66 distribution points were selected for analysis (Figure 1A).

The empirical investigation revealed that the flowering period of the *H. triflora* population in Haikou City extended from June to December, while the fruiting period spanned from June to January of the subsequent year. The duration of a single flowering event averaged 4.29 ± 0.85 days, with the stamen phase lasting 2.21 ± 0.64 days and the pistil phase persisting for 2.08 ± 0.95 days. The primary pollinator identified was *Xylocopa* sp. (Figure 1B). This species predominantly inhabited environments characterized by paddy fields, lakes, and wetlands (Figure 1C).

### 2.2. Environmental Parameters for Model Simulation

The climate data for the three historical periods of the LIG, LGM, and MH as well as the current period were obtained from WorldClim-Global Climate Data version 2.1 with a spatial resolution of 2.5 arc min [25]. The future climate data for the four time periods of 2021–2040, 2041–2060, 2061–2080, and 2081–2100 are based on three different climate scenarios (SSP-126, SSP-245, and SSP-585, SSP—shared socioeconomic pathways) of the global climate models (GCMs) of BCC_CSM2_MR. The slope and slope direction were extracted from DEM digital elevation data with an accuracy of 25 m, which were obtained from the Computer Network Information Center of the Chinese Academy of Sciences and the International Website of Scientific Data (http://www.gscloud.cn/, accessed on 5 March 2023).

ENMTools was used to conduct correlation analysis on 19 climate and environmental variables. When the correlation between two climate and environmental variables was greater than 0.8 [26,27], the climate and environmental factor with the greatest contribution to the influence on *H. triflora* distribution among the two climate and environmental variables was retained for subsequent analysis. A total of 12 environmental variables were used for analysis (Table 1), including 9 climate factors, namely, bio02, bio04, bio06, bio10, bio11, bio14, bio15, bio16, and bio19, and 3 terrain factors, namely, elevation, slope, and slope direction.

### 2.3. MaxEnt Model Description and Modeling

The MaxEnt model (version 3.4.1) was used to predict species distributions based on 66 global distribution records of *H. triflora* and 12 selected environmental variables. A total of 75% of the distribution data were randomly selected for training, and the remaining 25% of the distribution data were used for testing to determine the influence of each selected environmental factor on the *H. triflora* distribution. The jackknife test of variable importance was used. The area under the receiver operating characteristic (ROC) curve (AUC) was used to verify the accuracy of the model prediction.

The AUC is positively correlated with the prediction accuracy of the MaxEnt model and ranges from 0 to 1. When the AUC is less than 0.6, the prediction fails. When the AUC is greater than 0.6 and less than 0.7, the prediction accuracy is poor. When the AUC is greater than 0.7 and less than 0.8, the prediction is normal. When the AUC is greater than 0.8 and less than 0.9, the prediction accuracy is better. When the AUC is greater than 0.9, the prediction accuracy is very high [27,28].

### 2.4. Division of Potentially Suitable Areas

Based on the prediction results of the MaxEnt model, the Jenks natural breaks algorithm in ArcGIS 10.8 software was used to classify the suitable areas [29,30], and the suitability levels were divided into optimal areas, suitable areas, marginal areas, and unsuitable areas according to the probability of occurrence.

## 3. Results

### 3.1. MaxEnt Model Evaluations and Environmental Characteristics of H. triflora

**Figure 2:**
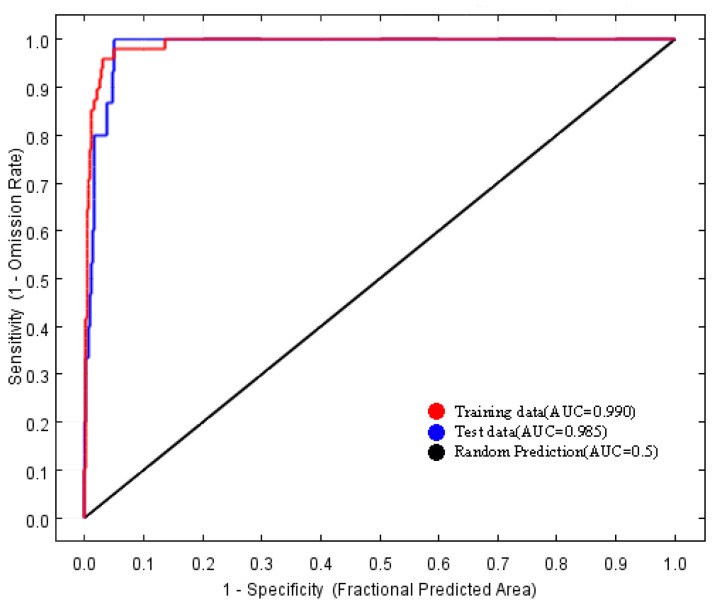
Receiver operating characteristic curve (ROC) of the MaxEnt models.

The area under the receiver operating characteristic (ROC) curve for the model was 0.990 (Figure 2), which exceeds the threshold of 0.90. Consequently, the predictive performance of the MaxEnt model is considered highly accurate and reliable. 

**Figure 3:**
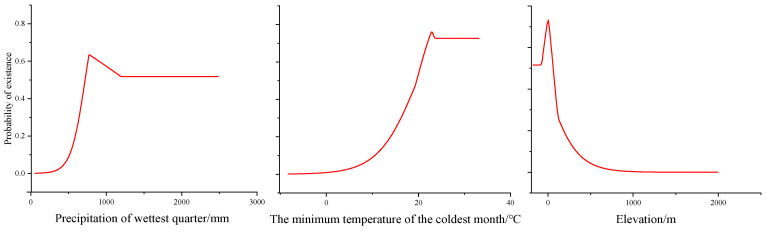
Response curves of existence probability of *H. triflora* for environmental variables.

Precipitation in the wettest quarter, the minimum temperature of the coldest month, and elevation are the main environmental variables that impact the geographical distribution of *H. triflora* (Table 2); their contribution rates are 36.10%, 24.30%, and 20.00%, respectively, and the total contribution rate of the above three environmental factors is 80.4%. The other variables have a combined contribution rate of only 19.6%. The combined contribution rate of the other variables is only 19.6%. The response curve of *H. triflora* to key environmental variables (Figure 3) indicates that, to achieve a probability exceeding 50%, the precipitation in the wettest quarter must be greater than 722.24 mm, the minimum temperature of the coldest month should be above 19.79 °C, and the elevation should be less than 50.58 m.

### 3.2. Historical Potential Distribution Area for H. triflora

**Figure 4:**
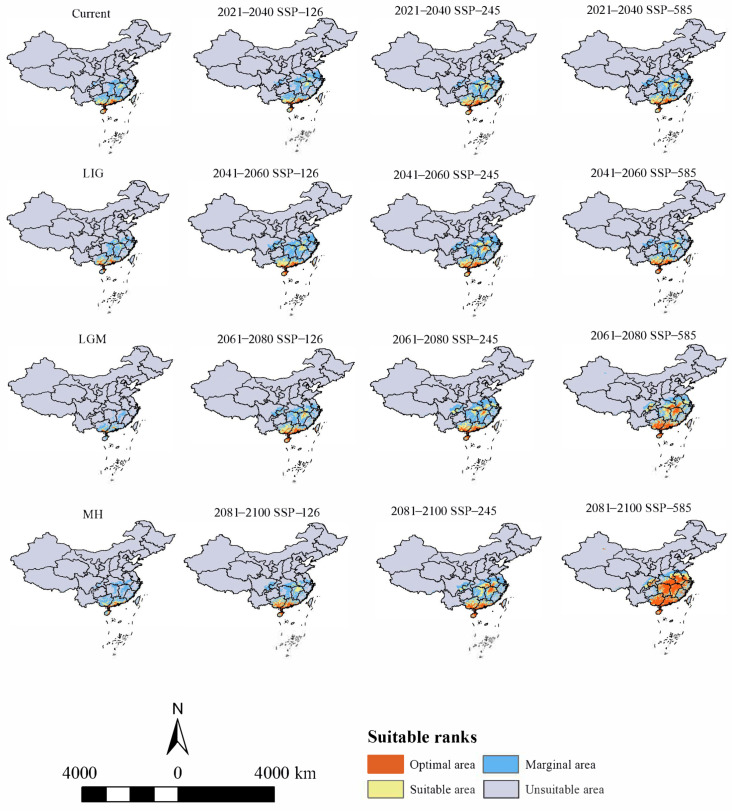
Distribution of potential suitable areas of *H. triflora* in historical, current, and future climate scenarios.

The potential distribution area of *H. triflora* in the past three periods was smaller compared to the current period (Figure 4). Notably, during the last interglacial (LIG) period, the potential distribution area reached its maximum extent of 61.14 × 10^4^ km^2^, predominantly located in southern Guangxi Province, central and southern Guangdong Province, and northeastern Hainan Province. In contrast, during the last glacial maximum (LGM), the potential distribution area contracted significantly to a minimum of 27.03 × 10^4^ km^2^, with the distribution primarily confined to the southern extremity of Guangxi Province and the central and southern regions of Guangdong Province. During the MH period, the potential distribution area expanded to 55.88 × 10^4^ km^2^, with the optimal habitat predominantly located in southwestern Guangdong Province and northeastern Hainan Province.

### 3.3. Current Potential Distribution Area for H. triflora

**Figure 5:**
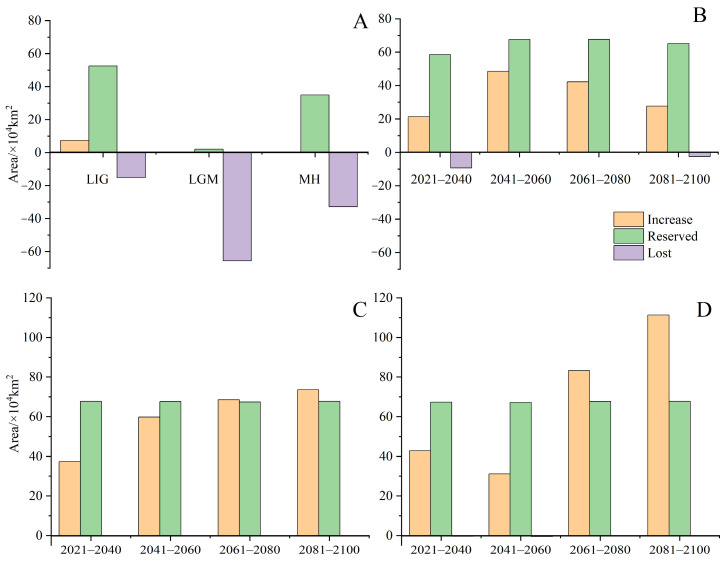
The changes in potential suitable area of *H. triflora* in China under historical, current, and future climate scenarios. ((**A**): The potential suitable area of *H. triflora*, historic and current; (**B**): The potential suitable area of *H. triflora* in SSP-126 climate scenario; (**C**): The potential suitable area of *H. triflora* in SSP-245 climate scenario; (**D**): The potential suitable area of *H. triflora* in SSP-585 climate scenario).

Under current climatic conditions (Figure 4), our findings indicate that the potential distribution area of *H. triflora* is primarily situated in the low-altitude regions of south-central China, southeastern China, and southern China, encompassing a total area of 67.81 × 10^4^ km^2^. This represents approximately 7.04% of China’s total land area. The optimal area is 8.45 × 10^4^ km^2^, accounting for 0.88% of China’s total land area. This area is predominantly located in Hainan Province, Guangdong and Guangxi provinces, and the northern region of Taiwan Province. In addition, Poyang Lake in Jiangxi Province is also a potential distribution area for *H. triflora* (Figure 4). Compared with those in the LIG, LGM, and MH periods, the potential distribution areas of *H. triflora* under the current climate scenarios have increased by 6.67, 70.78, and 11.93 × 10^4^ km^2^, respectively. However, the potential distribution areas have remained constant at 52.48, 2.11, and 34.94 × 10^4^ km^2^, respectively. The potential distribution areas of 15.23, 65.60, and 32.77 × 10^4^ km^2^ increased, and an area of 7.38, 0, and 0.38 × 10^4^ km^2^ decreased (Figure 5).

### 3.4. Changes in Future Potential Distribution Areas for H. triflora

**Figure 6:**
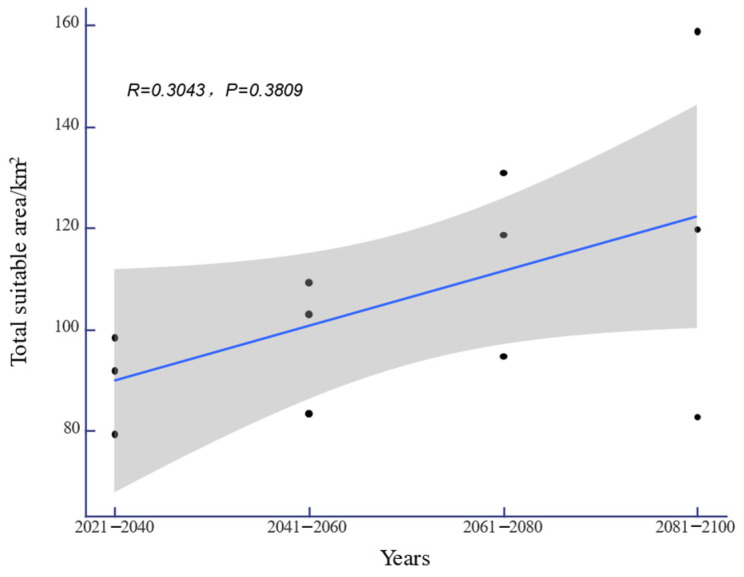
The changes in the adaptive area of *H. triflora* under future climate scenarios (the black dots indicate the suitable areas of *H. triflora* in different climate scenarios).

Under the SSP-126, SSP-245, and SSP-585 climate scenarios in the future, the potential distribution area of *H. triflora* in China is projected to gradually increase over time (Figure 6). The total potential distribution area will reach 79.08~158.64 × 10^4^ km^2^, with an overall increase of 11.27~90.83 × 10^4^ km^2^. The maximum potential distribution area, estimated at 158.64 × 10^4^ km^2^ (Figure 5), is expected to occur from 2081 to 2100 under the SSP-585 scenario. By 2100, the potential distribution area of *H. triflora* under the SSP-126, SSP-245, and SSP-585 climate scenarios will have increased, with the newly increased area ranging from 21.33 to 111.34 × 10^4^ km^2^ and the lost area below 9.29 × 10^4^ km^2^. During 2021–2040, the area of potential distribution area will decrease the most under the SSP-126 climate scenario (Figure 6). Compared to the current climate scenario, the potential distribution area of *H. triflora* is projected to expand under three future climate scenarios, although the trends in these changes will not be uniform. Under the SSP-126 climate scenario, the total potential distribution area of *H. triflora* will reach its peak during 2021–2040, exhibiting an initial increase followed by a decrease. However, the optimal habitat area will continue to expand. Specifically, regions such as Poyang Lake and the western side of Taiwan Province are anticipated to transition from suitable to optimal habitats, with the exception of Guangdong Province and Hainan Province. Under the SSP-245 climate scenario, the potential distribution area of *H. triflora* in the future will always increase. Under the SSP-585 climate scenario, the potential distribution area will first decrease and then increase and will peak from 2081 to 2100 (Figure 4). Under both the SSP-245 and SSP-585 climate scenarios, the optimal habitats are predominantly located in Hainan Province, south-central Guangdong Province, Guangxi Province, western Taiwan Province, the Poyang Lake basin, and the Yangtze River region at the junction of Jiangxi, Anhui, and Hubei provinces. Over time, the potentially suitable areas for *H. triflora* will gradually expand northward in Guangdong and Guangxi Provinces. At the same time, the optimal area of Poyang Lake expands to both sides of the Yangtze River.

### 3.5. Migration of the Distribution Centroid in the Potential Distribution Area under Different Periods

**Figure 7:**
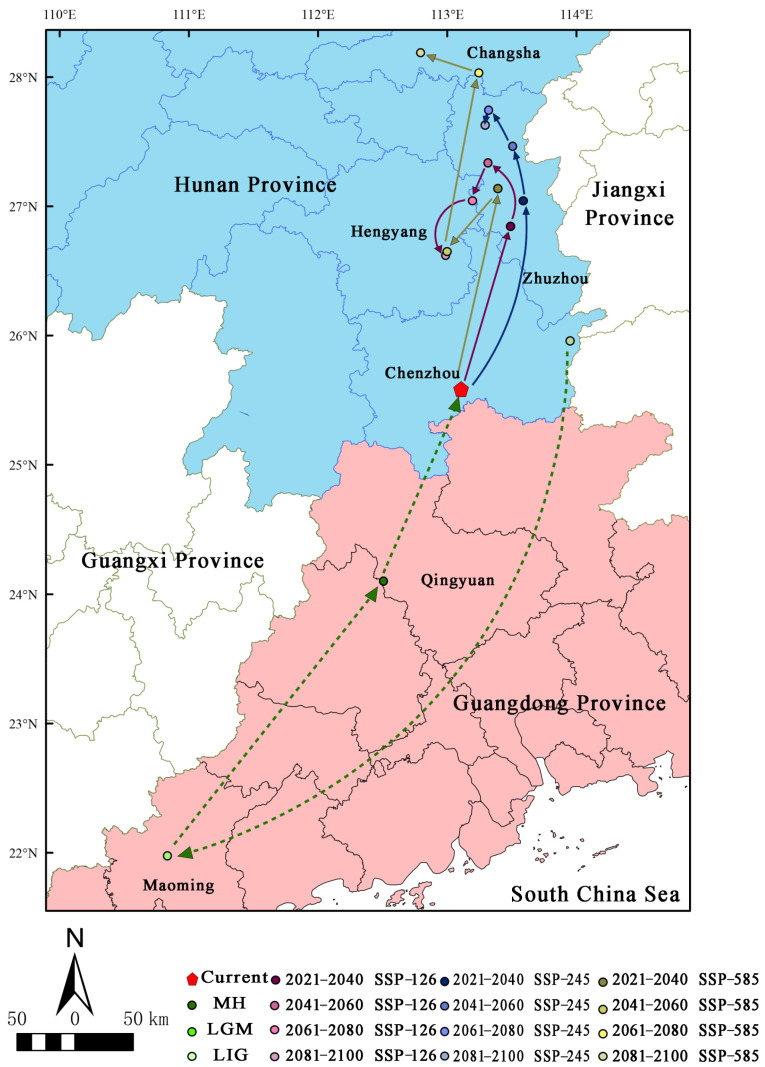
Distribution centroid of *H. triflora* under different climate change scenarios. (The green line indicates the historical migration path; the purple line indicates the migration path under SSP-126 climate change scenario in 2021–2100; the blue line indicates the migration path under SSP-245 climate change scenario in 2021–2100; the earthy yellow line indicates the migration path under SSP-585 climate change scenario in 2021–2100).

Under the current climate scenario (Figure 7), the distribution centroid of *H. triflora* is located in Chenzhou city, Hunan Province (113°29′21″ E, 26°50′37″ N), and during the LIG period, the distribution centroid is located in Chenzhou city, Hunan Province (113°57′7″ E, 25°57′28″ N). In contrast, during the LGM (Figure 7), the distribution centroid shifts 3.98° southward to Maming city, Guangdong Province (110°50′11″ E, 21°58′23″ N), representing a displacement of 473.862 km from the current centroid. During the MH period (Figure 6), the distribution centroid shifts 2.13° towards higher latitudes, relocating to Qingyuan City, Guangdong Province (112°30′32″ E, 24°5′55″ N), which is 93.837 km from the current distribution centroid. Under the three future climate scenarios (Figure 7), the distribution centroid of *H. triflora* is projected to shift further northward, especially by 2100, and under the SSP-585 scenario, the distribution centroid will shift 2.62° to higher latitudes, reaching its northernmost point, which is located in Changsha, Hunan Province (112°47′39″ E, 28°11′9″ N), migrating 299.992 km. Under the SSP-126 climate scenario, the distribution centroid will shift to higher latitudes, reaching the northernmost point in 2041–2060, which is located in Zhuzhou city, Hunan Province (113°18′56″ E, 27°20′4″ N), 203.261 km away from the current distribution centroid, and will gradually shift to lower latitudes in the next 40 years. In 2100, it is located in Hengyang city, Hunan Province (112°59′17″ E, 26°37′15″ N), at a distance of 121.278 km from the current distribution centroid. Under the SPS-245 climate scenario, the distribution centroid reaches the northernmost point of Zhuzhou city in Hunan Province (113°19′14″ E, 27°44′34″ N) during 2061–2080 and migrates 249.431 km to higher altitudes. Additionally, in Zhuzhou city (113°17′35″ E, 27°37′40″ N), the centroid moves 0.12° south, a distance of 236.215 km from its current distribution centroid.

## 4. Discussion

### 4.1. Model Accuracy Assessment

Predictions regarding the potential distribution of endangered species are becoming an integral component of conservation and management strategies [31,32,33]. Currently, among the various species distribution models available, the MaxEnt model is extensively utilized across multiple disciplines due to its benefits, including the ability to function with small sample sizes, ease of operation, and high accuracy [11,14,34,35]. The area under the curve (AUC) is one of the most frequently employed metrics for assessing the accuracy of the MaxEnt model [33]. The AUC quantifies the area under the ROC curve, which plots the true positive rate (sensitivity) against the false positive rate (1-specificity) at different threshold levels [36]. A higher AUC value corresponds to a lower overall omission rate and improved performance of the omission curve, signifying a better fit of the model to the prediction results [33,37,38]. In this study, the MaxEnt model was employed to predict the distribution of the endangered plant species *H. triflora* in potential distribution areas within China. Additionally, a jackknife test was conducted to assess the influence of each selected factor. The model achieved an AUC of 0.990, indicating that 99% of the training presences were accurately predicted, thereby demonstrating a high level of accuracy in the results.

### 4.2. Environmental Factors Limit the Distribution Area of H. triflora 

Temperature and precipitation are pivotal climatic determinants influencing species distribution [33,39]. Our findings indicate that precipitation during the wettest quarter, the minimum temperature of the coldest month, and elevation significantly impact the distribution of *H. triflora*. We employed the response curves of environmental factors to elucidate the relationship between potential habitat areas and environmental variables [30]. When the probability of species occurrence exceeds 50%, the corresponding environmental conditions are deemed conducive to plant growth [30,40]. Our study found that when the precipitation in the wettest quarter is greater than 722.24 mm, the minimum temperature of the coldest month exceeds 19.79 °C, and the elevation is below 50.58 m, the probability of the existence of *H. triflora* is greater than 50%. The best environmental conditions for survival are when the precipitation of the wettest quarter is 776.41 mm, the coldest month is 22.78 °C, and the elevation is 5.73 m. *H. triflora* is a thermophilic aquatic plant that also adapts to terrestrial habitats, thriving in consistently humid environments and requiring high levels of both water and temperature. The minimum temperature of the coldest month is a critical determinant for the winter survival of *H. triflora*. While elevation accounts for 20% of the species’ distribution, temperature decreases with increasing altitude also play a role [41]. Consequently, we posit that the impact of elevation on the potential distribution of *H. triflora* is primarily mediated by temperature. Furthermore, *H. triflora* predominantly inhabits aquatic environments where human activities are more prevalent, posing a significant constraint on population expansion.

### 4.3. Changes in Potential Distribution Area under Historic, Current, and Future Periods

The response of plants to climate change, driven by alternating ice ages and interglacial periods, is evidenced through geographical distribution migration and adaptive evolution [42,43]. During the LIG period, the average land temperature was 1–2 °C higher than current levels, resulting in a climate environment relatively analogous to the present [44]. Consequently, more suitable habitats for *H. triflora* emerged, with the primary potential distribution areas closely resembling those identified in the current study. With the arrival of the LGM, the potential distribution areas of *H. triflora* contracted southward, and the distribution centroid migrated to lower latitudes. This shift is likely attributable to the LGM being the coldest and driest period since the Quaternary [45]. During this epoch, global average temperatures were 5–12 °C lower than present-day levels [43], which may have significantly contributed to the substantial reduction in the potential distribution area of *H. triflora*. Simultaneously, extensive portions of the land surface were enveloped by glaciers during this epoch, with glacial coverage in China expanding by a factor of 8.7 compared to the present period [43]. This glaciation posed additional challenges to the survival of *H. triflora*. With the onset of the MH period, temperatures began to rise gradually [46], resulting in an average land temperature in China that was 1–1.5 °C higher than current levels [47]. Consequently, the potential distribution areas of *H. triflora* progressively extended to higher latitudes, and the species’ distribution centroid in China shifted northward.

Currently, the suitable and optimal habitats for *H. triflora* are primarily located in the coastal regions of Hainan Island, the western coastal zone of Taiwan Island, the Leizhou Peninsula, the middle and lower reaches of the Pearl River, and the Poyang Lake basin. Nonetheless, *H. triflora* has only been documented on Hainan Island, likely due to the Qiongzhou Strait acting as a natural geographical barrier, impeding its dispersal to mainland areas. Similar phenomena are observed in other regions; for example, *Cacaiao melanocephalus* and *Pithecia pithecia* are confined to either side of the Rio Blanco River and cannot spread to the other side in Guyana [48]. *Metapetrocosmea peltata*, which is distributed on both sides of the Changhua River Valley, experiences restricted gene flow due to the river valley acting as a barrier in Hainan. This results in significant genetic differentiation between the two populations [49].

In the future, with anticipated changes in global climate, the potential distribution range of *H. triflora* is projected to expand beyond its current geographical confines, with the centroid of its distribution shifting towards higher latitudes. However, *H. triflora* will predominantly remain concentrated in the southern regions of China. Under the SSP-126, SSP-245, and SSP-585 climate scenarios, the extent of potentially suitable habitats for *H. triflora* is expected to increase to varying degrees, with an increasing trend from SSP-126 to SSP-245, and the highest increase under SSP-585. This phenomenon can be attributed to the SSP-585 scenario being a high radiative forcing scenario with an average temperature increase of 2.4~4.8 °C by 2100. In contrast, the SSP-126 scenario has a temperature increase of less than 2.0 °C, and SSP-245 is between SSP-126 and SSP-585 [50]. Consequently, the SSP-585 scenario would provide more favorable hydrothermal conditions for *H. triflora*, whereas the SSP-126 scenario would offer the least favorable conditions among the three climate scenarios. The potential habitable area of *H. triflora* reaches a maximum of 158.64 × 10^4^ km^2^ under the SSP-585 climate scenario in 2100. Under the most extreme SSP-585 climate scenario, the suitable and optimal areas for *H. triflora* are projected to expand significantly towards higher latitudes compared to current conditions. Additionally, a limited number of low-altitude regions in the provinces of Yunnan, Xinjiang, Sichuan, Chongqing, and Tibet have also emerged as optimal areas. This expansion is likely attributable to global warming, which has resulted in increased temperature and precipitation in these regions, thereby rendering them suitable for *H. triflora*. These newly identified optimal areas will offer new distribution zones with appropriate field environments for the introduction and conservation of the species.

*H. triflora* is predominantly found in rice paddies, wetlands, and other aquatic habitats with significant human activity in Hainan Province. It is commonly used as pig feed and is extensively harvested, potentially leading to a decline in its population. In addition, *H. triflora* habitat can also be compressed by urbanization, man-made landscapes, and other anthropogenic factors. This situation necessitates governmental intervention to raise public awareness and guide residents in strengthening the protection of wild populations. To better understand *H. triflora*’s main distribution areas, we need to identify key functional zones, conduct detailed habitat surveys, investigate factors affecting its distribution and growth, and collect germplasm resources for a comprehensive database. Concurrently, the establishment of local conservation areas is a crucial protective measure that will support the recovery and expansion of wild populations.

## 5. Conclusions

In this study, the MaxEnt model was used to predict the past, present, and future potential habitat area of *H. triflora*. The main results show that: (i) The main environmental variables affecting the distribution of *H. triflora* are precipitation in the coldest month and in the wettest quarter, and elevation is also a main factor. (ii) In the three past periods of LIG, LGM, and MH, the suitable area of *H. triflora* tended to decrease first and then increase, and the suitable area reached the lowest value in the LGM period, at only 27.03 × 10^4^ km^2^. Southern Guangxi and southern Guangdong were optimal areas in the three past periods. The centroid of the species distribution in the LIG, LGM, and MH shifted first to low latitudes and then to high latitudes. (iii) The current potential distribution area is 67.81 × 10^4^ km^2^, accounting for 7.04% of China, and it is mainly distributed in southern China, of which the optimal area is mainly distributed in Guangxi, Guangdong, and Hainan provinces. (iv) Under the future climate scenario, the potential distribution area of *H. triflora* increases by 11.27~90.83 × 10^4^ km^2^ under the three climate scenarios and reaches a maximum value (158.64 × 10^4^ km^2^) from 2081 to 2100 under the SSP-585 climate scenario, and the distribution centroid of *H. triflora* shifts to higher latitudes.

## Figures and Tables

**Table 1:** Contribution of variables for modeling.

Variable	Description	Percent Contribution/%
bio02	Mean diurnal range	3.9
bio04	Variation in temperature seasonality	0.9
bio06	Minimum temperature of the coldest month	24.3
Bio10	Mean temperature of warmest quarter	4.5
Bio11	Mean temperature of coldest quarter	3.9
Bio14	Precipitation of the driest month	0.6
Bio15	Variation in precipitation seasonality	3.4
Bio16	Precipitation of wettest quarter	36.1
Bio19	Precipitation of coldest quarter	3.2
Elev	Elevation position of species	20.0
Slope	Slope position of species	1.5
Aspect	Aspect position of species	1.0

**Table 2:** The potential distribution area of *H. triflora* in China under historical, current, and future climate scenarios (×10^4^ km^2^).

Climate Scenario	Year	Optimal Area	Suitable Area	Marginal Area	Unsuitable Area
	LIG	7.43	13.79	39.92	902.71
	LGM	1.60	4.33	21.10	936.82
	MH	4.77	9.81	41.30	907.97
	Current	8.45	16.11	43.25	896.04
SSP-126	2021–2040	8.83	15.36	54.89	884.82
SSP-126	2041–2060	10.81	23.86	68.04	861.14
SSP-126	2061–2080	13.49	24.34	56.64	869.38
SSP-126	2081–2100	13.46	19.97	49.03	881.39
SSP-245	2021–2040	13.93	25.64	52.02	872.26
SSP-245	2041–2060	15.56	28.11	65.36	854.82
SSP-245	2061–2080	15.86	34.56	67.95	845.47
SSP-245	2081–2100	22.72	35.27	61.51	844.35
SSP-585	2021–2040	12.34	25.28	60.46	865.76
SSP-585	2041–2060	14.90	20.32	47.92	880.71
SSP-585	2061–2080	34.05	44.35	52.29	833.15
SSP-585	2081–2100	75.94	45.97	36.72	805.21

## Data Availability

Species occurrence records were obtained from the Global Biodiversity Information Facility database (GBIF: https://www.gbif.org/, accessed on 3 March 2023), the Plant Photo Bank of China (PPBC: http://ppbc.iplant.cn/, accessed on 3 March 2023), the National Specimen Information Infrastructure database (NSII: http://nsii.org.cn/2017/home.php, accessed on 3 March 2023), and our field surveys.
